# Supplementary material for: Evaluating the Practices and Challenges of Youth Volleyball Development in Amhara Regional State, Ethiopia by Using the CIPP Model
Source: Healthcare (Basel). 2022 Apr 13;10(4):719. doi: 10.3390/healthcare10040719 (PMC9031901; doi:10.3390/healthcare10040719)
Supplement: Supplementary file 1 [file healthcare-10-00719-s001.zip › healthcare-1591939-supplementary.pdf]

S1: The open-ended items in a youth volleyball player's survey questionnaire

1. What is your overall perception about the existing youth volleyball program at Amhara regional state?

2. What is your comment about the existing youth volleyball program at Amhara Regional State?

S2: Semi-structured Interview Guide

Interview questions

1. How is the current youth volleyball development program operating in Amhara regional state? Did you develop guidelines and strategies designed to fulfill the necessary resources and facilitate the implementation process?
2. What forms of support (social and material) and monitoring and follow-ups your office and other responsible offices have given for the youth volleyball development program in Amhara regional state? How do you comment on the role of family, schools, and media in the program implementation?
3. In your view, how do you comment about the effectiveness and impacts of the youth volleyball development program in the Amhara regional state?
4. What is the facilitating and hindering contextual factors associated with the implementation of the youth volleyball development program in the Amhara regional state? What are the key challenges of the program in Amhara regional state?
5. What is your overall comment about the youth volleyball development program in the Amhara regional state?

S3: Guide for an FGD with Youth Volleyball Coaches

Discussion Points

1. The youth volleyball development program implementation in Amhara regional state, Ethiopia.
   - Comments on proper implementation.
   - Comments on the facilitation by the resources and support mechanisms?
   - The main obstacles hindering the implementation of the program.
2. Evaluate the youth volleyball development program in Amhara regional state.
   In terms of the youth coaches' behavior and training delivery
   - The scientific method of training
   - Training capacity
   - Implementation according to the training plan
   - professional development opportunities

   From the youth players and the training context
   - Convenience of the project site and the training field
   - Use of suitable equipment and materials for training
   - Attending the training on time
   - Ability to quickly apply learned materials during the training
3. How do you evaluate the effectiveness and impacts of the youth volleyball development program in the Amhara regional state?
   - Effectiveness of the program in meeting its targets.
   - The impacts for the youth and overall development of volleyball in the region and nationally.
4. The level of commitment and support for the proper implementation of the youth volleyball program in Amhara regional state.
   - The regional volleyball federation
   - The leadership of the regional sports administration
   - MoE and High schools
   - Family
   - Media
5. Overall comment about the youth volleyball development program in Amhara regional state.
   - Contextual factors
   - Key challenges
   - Suggestions for improvement

Many thanks for your time and feedback!!!
